# Analysis of diurnal to seasonal variations and trends in air pollution potential in an urban area

**DOI:** 10.1038/s41598-023-48420-x

**Published:** 2023-11-29

**Authors:** Mahshad Soleimanpour, Omid Alizadeh, Samaneh Sabetghadam

**Affiliations:** Institute of Geophysics, University of Tehran, Tehran, Iran

**Keywords:** Climate sciences, Atmospheric science, Climate change

## Abstract

Air pollution is the world’s largest environmental health threat to humans and has wide-ranging adverse effects on the environment. The term ventilation coefficient (VC), which is a function of the average wind speed through the planetary boundary layer (PBL) and the PBL height (PBLH), can be used to estimate air pollution potential. We analyzed PBLH, wind speed through PBL, and VC over Tehran using ERA5, and PM2.5 surface concentration using MERRA-2 during 1991–2020. Both PBLH and VC undergo substantial diurnal variations, with higher values during the day and much lower values at night. As a result, PM2.5 concentration in Tehran is the maximum in the early morning, while it is relatively lower in the afternoon. The average wind speed through PBL shows the same diurnal variation in all seasons, except in winter when winds in PBL are stronger at night than during the day. Both PBLH and VC over Tehran show substantial seasonal variations, with much higher values in summer followed in decreasing order by spring, autumn, and winter, highlighting an extremely high air pollution potential in winter. Hence, due to high pollutant emissions, the occurrence of severe air pollution is expected to be a common feature in Tehran in winter. PBLH has significantly increased over Tehran both during the day and at night for the period 1991–2020, primarily in response to the surface warming in recent decades, while wind speed through PBL has significantly declined only at night. The overall impact of such changes is an increase in VC over Tehran both during the day and at night, although the increasing trend of VC is statistically significant only at night. Our results highlight the urgent need for the implementation of effective sustainable policies to reduce air pollution and its adverse effects in winter when air pollution potential is high in Tehran.

## Introduction

Air pollution is considered the world’s largest environmental health risk to humans, causing approximately 9 million premature deaths each year, corresponding to one in six deaths globally^1^. It refers to the release of a mixture of gaseous and particulate pollutants emitted from both natural and anthropogenic sources^2^. Air pollution is primarily the unintended consequence of industrialization, urbanization, and fast economic growth^3^. Depending on the existence of sources of air pollutants^4^ and the dominant atmospheric conditions^5,6^, the degradation of air quality is regionally different^7^. Air pollution is particularly a major issue in developing countries^8,9^ where the emission of air pollutants is larger^7^ because a low priority is given to environmental pollution. However, air pollution is also largely controlled by atmospheric conditions^5,6^, such that severe air pollution episodes generally occur in areas where the ventilation of air pollution is poor [e.g.^10,11^]. Irrespective of the existence of sources, the degree of the ventilation of air pollutants by atmospheric conditions is defined as air pollution potential^12^.

Planetary boundary layer height (PBLH) and the average wind speed through the planetary boundary layer (PBL) are the main meteorological factors that determine the ventilation of air pollutants in the vertical and horizontal directions, respectively^6,13^, the product of which is defined as the ventilation coefficient (VC)^14^. VC has been widely used to determine air pollution potential in different regions across the globe^12,14^. Higher values of PBLH and the average wind speed through PBL lead to higher values of VC, which imply good ventilation of air pollutants and a low potential for the accumulation of air pollutants.

Several studies have already been conducted to determine air pollution potential in different areas [e.g.^6,12,13^]. For example, the results of Azargoshasbi et al.^11^ indicate that a reduction in both VC and PBLH was associated with a severe air pollution episode in Tehran. Holzworth^12^ applied the mixing layer depth and the average wind speed through the mixed layer in an urban diffuse model to obtain concentrations of air pollutants over four different cities in the United States. Their results indicate that the concentration of air pollutants is lower in the afternoon than in the morning for all cities. Based on the analysis of the near-surface wind speed and PBLH, Gassmann and Mazzeo^13^ concluded that air pollution potential is relatively high in northeastern and central-eastern Argentina all year long. The results of Rigby et al.^15^ indicate that under southeasterly winds, air pollution increases in many areas in the United Kingdom (UK). They argue that southeasterly winds are relatively weak and advect high-level warm air toward the UK, which contributes to the stabilization of the PBL and a reduction in PBLH. By the analysis of VC over Kochi city in India, Goyal and Chalapati Rao^5^ found that the ventilation of air pollutants is high in the afternoon, but reduces in the morning and evening. Lu et al.^6^ analyzed the diurnal variation of VC over Changsha in China and found higher values and a larger variation of VC during the day, but lower values and relatively constant values of VC at night. The analysis of Sujatha et al.^16^ indicates that the maximum VC over Hyderabad in India is during summer, while the minimum is in winter. Iyer and Raj^17^ indicated that VC decreased over four major metropolitan cities of India in winter during the period 1971–2000, while the results of Saha et al.^18^ indicate that VC increased by the rate of 70 m$^2$ s$^{-1}$per year over Delhi during the period 2006–2014.

Following rapid population growth, urban expansion, and industrialization, air pollution has become a major environmental threat in Tehran in recent decades^9,19,20^. Indeed, Tehran is one of the most polluted cities in the Middle East with a high premature mortality rate^21^, yet there is a lack of investigation into the identification of air pollution potential in this city. Hence, this study aims to analyze PBLH, the average wind speed through PBL, and VC over Tehran on diurnal, monthly, and seasonal timescales, and to determine whether these boundary layer features have undergone any long-term significant trends. To this end, PBLH and the average wind speed through PBL were obtained from the European Centre for Medium-Range Weather Forecasts (ECMWF) Reanalysis 5th Generation (ERA5) data, based on which VC has been calculated and analyzed on the abovementioned timescales. Our results highlight an extremely high air pollution potential over Tehran in winter, which under the existence of pollutant emissions, is conducive to the occurrence of severe air pollution episodes. VC over Tehran also shows a significant increasing trend at night during the period 1991–2020.

## Data description and methodology

**Figure 1:**
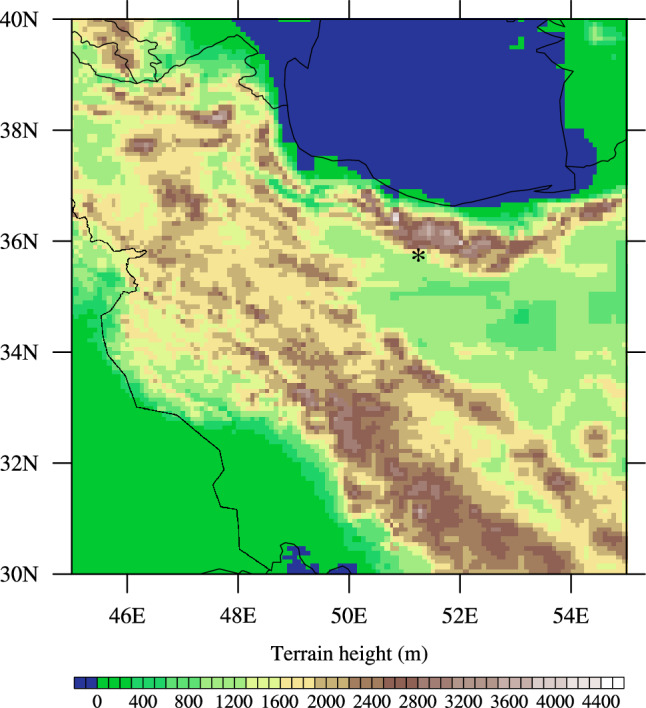
The approximate geographic location of Mehrabad meteorological station (35.75$^\circ$ N, 51.25$^\circ$ E, star) in Tehran, over which the ERA5 data were obtained and analyzed. The elevation (m) of the surrounding regions is also shown. Elevation data was taken from earth topography five minute grid (ETOPO5) (http://www.ngdc.noaa.gov/mgg/global/relief/ETOPO5/TOPO/ETOPO5/) and the NCAR Command Language version 6.6.2 was used to generate the figure (http://www.ncl.ucar.edu/).

We used the monthly ERA5 data^22^ with a horizontal resolution of $0.25^\circ \times 0.25^\circ$ during the period 1991–2020. The data that we used include zonal and meridional components of wind speed in different vertical levels through PBL and PBLH over Tehran (35.75$^\circ$ N, 51.25$^\circ$ E), the geographic location of which is shown in Fig. 1. We then derived wind speed in each vertical level from a square root of the sum of the squared of the zonal and meridional wind components, while VC was obtained by the product of PBLH and the average wind speed through PBL^14^. The lower values of VC imply a higher concentration of air pollutants or a higher air pollution potential and vice versa.

The ERA5 data, including zonal and meridional components of wind speed, are available at different pressure levels. As such, we have applied the hydrostatic equation to derive pressure values equivalent to PBLH at different times during the period 1991–2020, based on that only an average each time is taken for wind speeds in vertical pressure levels that are located below or equal to the pressure level equivalent to PBLH. To do the conversion using the hydrostatic equation ($\frac{dP}{dZ}=-\rho$g), air density ($\rho$) is considered a constant value of 1.293 kg m$^{-3}$ through PBL, the gravity acceleration (g) is considered 9.8 m$^{2}$ s$^{-2}$, and surface pressure values were obtained from the ERA5 data for the period 1991–2020. In addition, we obtained hourly and monthly means of surface PM2.5 concentration over Tehran from the Modern-Era Retrospective Analysis for Research and Applications, Version 2 (MERRA-2), which has a horizontal resolution of $0.5^\circ \times 0.625^\circ$^23^. The results of previous studies indicate that the MERRA-2 surface PM2.5 concentrations are less than ground-based observations [e.g.^24^]. Nevertheless, we used this dataset because continuous hourly ground-based PM2.5 observations are not available over Tehran for the period 1991–2020.

In this study, the split of the year into four seasons coincides with the Gregorian calendar, such that winter, spring, summer, and autumn are considered as December–January–February (DJF), March–April–May (MAM), June–July–August (JJA), and September–October–November (SON). We used a simple linear regression method for the trend analysis and tested the significance of trends using the classical t-test. For the diurnal analysis, the Universal Time Coordinated (UTC) is converted into local time (LT) in Tehran by adding 3:30 hours. All the hours mentioned in the diurnal analysis subsection are based on local times. In addition to the diurnal analysis, PBL features are analyzed at 00:00 UTC (03:30 LT) and 12:00 UTC (15:30 LT), which represent PBL features at night and during the day, respectively.

## Results

### Monthly and seasonal variations

**Figure 2:**
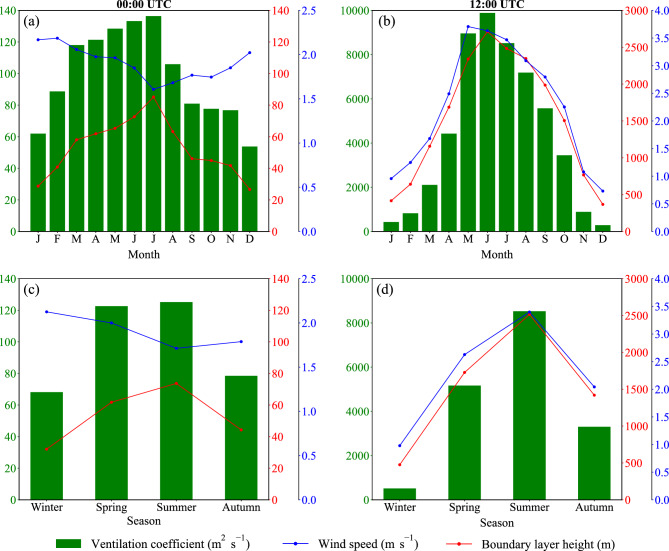
(**a**, **b**) Monthly and (**c**, **d**) seasonal variations of the planetary boundary layer height (PBLH, m, red lines), the average wind speed (m s$^{-1}$ ) through the planetary boundary layer (PBL) (blue lines), and the ventilation coefficient (VC, m $^{2}$ s$^{-1}$, green bars) over Tehran (35.75$^\circ$ N, 51.25$^\circ$ E) at (left panels) 00:00 UTC and (right panels) 12:00 UTC averaged during the period 1991–2020. We used Python 3.10.9 to generate the figure (https://www.python.org/downloads/release/python-3109/).

Fig. 2 shows long-term averages of PBLH, the average wind speed through PBL, and VC over Tehran in different months and seasons both at night (00:00 UTC, 03:30 LT) and during the day (12:00 UTC, 15:30 LT). At night, the maximum PBLH over Tehran reaches 85 m in July, while the minimum occurs in December (27 m) (Fig. 2a). As expected, PBLH is much higher during the day compared to night in all seasons. The maximum PBLH over Tehran occurs in June (2713 m) during the day (Fig. 2b) in response to high surface temperatures, corresponding to a highly turbulent atmospheric boundary layer. In contrast, the minimum PBLH during the day occurs in December (368 m) (Fig. 2b) when the surface temperature is relatively low, corresponding to the suppression of turbulence.

**Figure 3:**
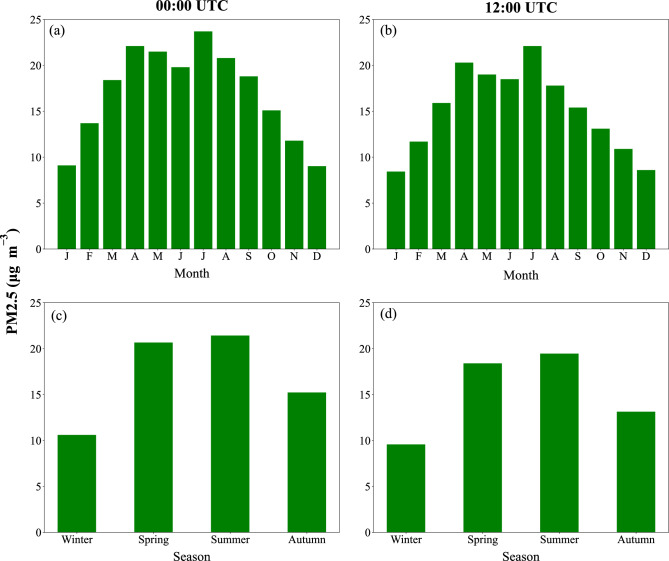
(**a**, **b**) Monthly and (**c**, **d**) seasonal variations of PM2.5 concentration ($\mu$g m$^{-3}$) over Tehran (35.75$^\circ$ N, 51.25$^\circ$ E) at (left panels) 00:00 UTC and (right panels) 12:00 UTC averaged during the period 1991–2020. We used Python 3.10.9 to generate the figure (https://www.python.org/downloads/release/python-3109/).

At night, the maximum and minimum wind speeds averaged through PBL occur in February (2.2 m s$^{-1}$) and July (1.6 m s$^{-1}$) (Fig. 2a), while the daytime maximum and minimum wind speeds averaged through PBL are in May (3.7 m s$^{-1}$) and December (0.7 m s$^{-1}$), respectively (Fig. 2b). Hence, while the maximum wind speed through PBL during the day occurs in summer, the maximum at night is in winter, implying that seasonal variation in wind speed when averaged during the whole day is relatively small. The maximum and minimum VC values over Tehran at night are in July (136 m$^{2}$ s$^{-1}$) and December (54 m$^{2}$ s$^{-1}$), respectively (Fig. 2a). In other words, air pollution potential at night is the highest and lowest in Tehran in December and July, respectively. The maximum and minimum VC values over Tehran during the day are in June (9885 m$^{2}$ s$^{-1}$) and December (284 m$^{2}$ s$^{-1}$), respectively (Fig. 2b). In other words, air pollution potential during the day is the highest and lowest over Tehran in December and June, respectively. Despite that, both at night and during the day, the minimum and maximum PM2.5 concentrations over Tehran occur in December and July, respectively (Fig. 3a,b).

Our analysis on the seasonal timescale indicates that both the maximum PBLH (Fig. 2c) and PM2.5 concentration (Fig. 3c) over Tehran at night occur in summer, followed in decreasing order by spring, autumn, and winter, with PBLH values of 74, 62, 44, and 32 m, respectively. A warmer land surface in summer nights compared to the other seasons largely contributes to the development of a higher PBL through buoyancy production^25^, although due to longwave radiative cooling of the surface at night, there is relatively small seasonal variation in PBLH at night. The maximum wind speed at night occurs in winter (2.1 m s$^{-1}$), followed in decreasing order by spring (2.0 m s$^{-1}$), autumn (1.8 m s$^{-1}$), and summer (1.7 m s$^{-1}$) (Fig. 2c). As VC is the product of PBLH and the average wind speed through PBL, the maximum VC over Tehran at night occurs in summer (125 m$^{2}$ s$^{-1}$) and spring (123 m$^{2}$ s$^{-1}$), followed in decreasing order by autumn (78 m$^{2}$ s$^{-1}$) and winter (68 m$^{2}$ s$^{-1}$) (Fig. 2c). Hence, there is a high potential for the accumulation of air pollutants over Tehran in all seasons at night, particularly in winter.

The maximum PBLH (Fig. 2d) and PM2.5 concentration (Fig. 3d) over Tehran during the day occur in summer, followed in decreasing order by spring, autumn, and winter, with PBLH values of 2515, 1728, 1419, and 476 m, respectively. Strong surface heat fluxes associated with the land surface warming in summer days contribute to the development of a highly turbulent PBL through buoyancy production^25^, which corresponds to an increase in PBLH. In contrast, PBLH is relatively low in winter due to surface cooling and the suppression of vertical mixing by stable stratification. The average wind speed through PBL over Tehran is the highest in summer during the day (3.4 m s$^{-1}$) in response to a highly turbulent PBL, followed in decreasing order by spring (2.6 m s$^{-1}$), autumn (2.0 m s$^{-1}$), and winter (1.0 m s$^{-1}$) (Fig. 2d). As VC is the product of PBLH and the average wind speed through PBL, the maximum VC over Tehran occurs in summer during the day (8529 m$^{2}$ s$^{-1}$) (Fig. 2d), which is indicative of the low potential for the accumulation of air pollutants compared to the other seasons. The ventilation coefficient is also relatively high in spring during the day (5164 m$^{2}$ s$^{-1}$) and autumn (3302 m$^{2}$ s$^{-1}$). In contrast, the minimum VC during the day occurs in winter (512 m$^{2}$ s$^{-1}$) (Fig. 2d), indicating the high potential for the accumulation of air pollutants. Note a large difference between the daytime VC in summer and winter, suggesting that air pollution potential over Tehran during the day substantially increases from summer to winter. Note also in Fig. 2c,d that in contrast to the maximum wind speed during the day that occurs in summer, the maximum wind speed at night is in winter. Hence, in contrast to PBLH and VC, seasonal variation in the average wind speed through PBL is relatively small because relatively stronger winds at winter nights compared to the other seasons partially compensate for the stronger winds during the day in warmer months of spring, summer, and autumn. Note that despite the high air pollution potential in winter compared to summer over Tehran (Fig. 2), PM2.5 concentration is much higher in summer than winter (Fig. 3) because Tehran is largely influenced by dust events in late spring and during summer^26^.

### Diurnal variation

**Figure 4:**
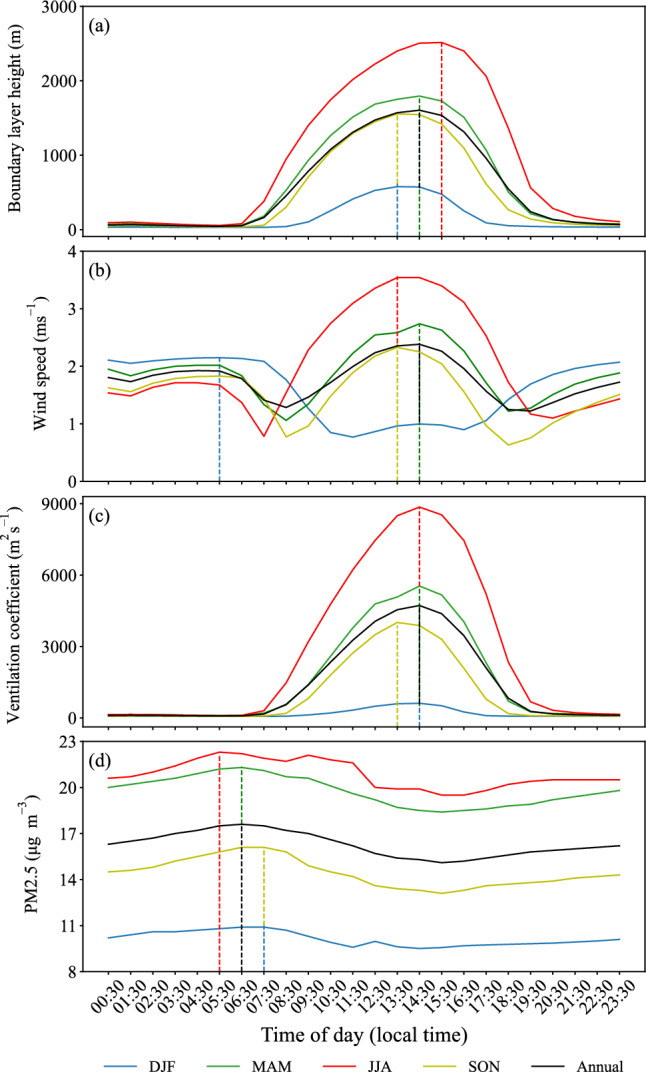
Diurnal variations of (**a**) the planetary boundary layer height (PBLH, m), (**b**) the average wind speed (m s$^{-1}$) through the planetary boundary layer (PBL), (**c**) the ventilation coefficient (VC, m$^{2}$ s$^{-1}$), and (**d**) PM2.5 concentration ($\mu$g m$^{-3}$) over Tehran (35.75$^\circ$ N, 51.25$^\circ$ E) in different seasons and annually averaged during the period 1991–2020. Vertical dashed lines indicate the time of occurrence of the maximum of each variable in different seasons and annually. Local time in Tehran is 03:30 hours ahead of UTC. We used Python 3.10.9 to generate the figure (https://www.python.org/downloads/release/python-3109/).

Figure 4 shows diurnal variations of PBLH, the average wind speed through PBL, VC, and PM2.5 concentration over Tehran in different seasons and annually based on long-term averages over the period 1991–2020. The maximum PBLH over Tehran in winter (577 m) occurs at 13:30 LT, while it substantially increases but also occurs later in the afternoon in relatively warmer seasons of spring (1795 m at 14:30 LT) and summer (2515 m at 15:30 LT). Similar to winter, the maximum PBLH (1555 m) occurs at 13:30 LT in autumn, but its value is approximately three times higher than in winter. Annually, PBLH over Tehran reaches the maximum value of 1605 m at 14:30 LT. The maximum average wind speed through PBL over Tehran in winter occurs early in the morning (05:30 LT), while it occurs in the afternoon (between 13:30 and 14:30 LT) in the other seasons and annually. This implies that the contribution of wind speed to the ventilation of air pollutants is larger early in the morning than in the afternoon in winter, although PBLH is larger in winter afternoons. The maximum VC over Tehran occurs in the early afternoon at 14:30 LT in winter (614 m$^{2}$ s$^{-1}$), spring (5539 m$^{2}$ s$^{-1}$), and summer (8855 m$^{2}$ s$^{-1}$) seasons, and annually (4722 m$^{2}$ s$^{-1}$), and at 13:30 LT in autumn (4010 m$^{2}$ s$^{-1}$). As a result, PM2.5 concentration in Tehran is relatively low in the afternoon (Fig. 4d).

Minimum values of both PBLH and VC over Tehran occur in the early morning between 05:30 and 07:30 LT in all seasons and annually. Hence, air pollution potential over Tehran is the worst in all seasons during these early morning hours (Fig. 4c), contributing to the maximum PM2.5 concentration (Fig. 4d). The minimum wind speed over Tehran also occurs in the morning in winter (11:30 LT), spring (08:30 LT), and summer (07:30 LT), but in the late afternoon in autumn (18:30 LT) and annually (19:30 LT) (Fig. 4). Note in Fig. 4 that the diurnal variation in both PBLH and VC over Tehran is much higher in summer, spring, and autumn than in winter (particularly in summer). In other words, substantial ventilation of air pollutants is expected to occur over Tehran during the day in warmer months of summer, spring, and autumn. In contrast, Tehran is characterized by poor ventilation of air pollutants both during the day and at night in winter, implying that air pollutants can be well accumulated on a relatively shallow PBLH over the course of the day and night.

### Interannual variation

**Figure 5:**
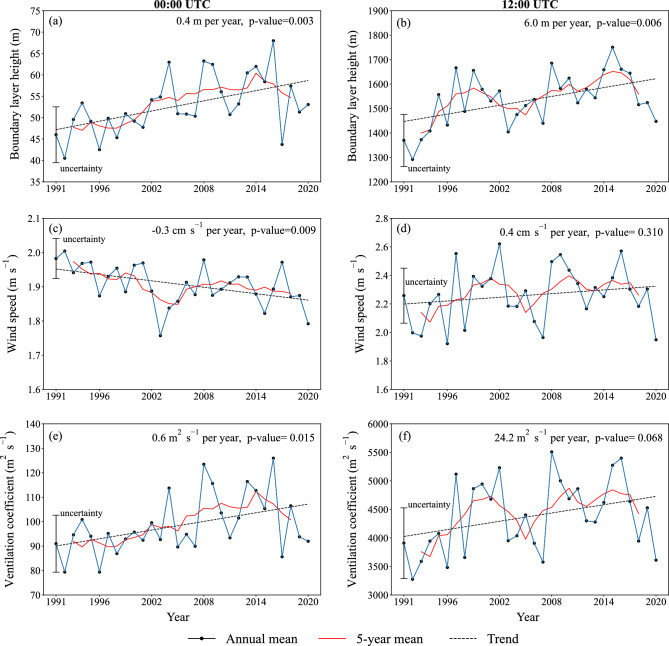
Time series of the annual means (solid blue lines) and 5-year running annual means (solid red lines) of the (**a**, **b**) planetary boundary layer height (PBLH, m), (**c**, **d**) the average wind speed (m s$^{-1}$) through planetary boundary layer (PBL), and (**e**, **f**) the ventilation coefficient (VC, m$^{2}$ s$^{-1}$) over Tehran (35.75$^\circ$ N, 51.25$^\circ$ E) during the period 1991–2020 at (left panels) 00:00 UTC (03:30 local time) and (right panels) 12:00 UTC (15:30 local time). Dashed lines indicate linear trends based on a simple linear regression method. Vertical lines show the uncertainty, which is obtained based on the standard deviation of each variable during the period 1991–2020. We used Python 3.10.9 to generate the figure (https://www.python.org/downloads/release/python-3109/).

Figure 5 shows the time series of the annual mean and 5-year running annual mean of PBLH, the average wind speed through PBL, and VC over Tehran for the period 1991–2020. PBLH has significantly increased over Tehran both at night (*p*-value = 0.003) and during the day (*p*-value=0.006), with a rate of 0.4 and 6.0 m per year, respectively (Fig. 5a,b). This could be caused by a substantial increase in the surface temperature of Tehran in recent decades, as also evident based on observations^27^, which contributes to an increase in surface heat fluxes. Wind speed through PBL at night has significantly declined ($-\,$0.3 cm s$^{-1}$, *p*-value = 0.009) (Fig. 5c), which partially compensates for the ventilation of air pollutants in PBL due to an increase in PBLH (Fig. 5a). However, the impact of the increase in PBLH is stronger because VC over Tehran at night has significantly increased at a rate of 0.6 m$^{2}$ s$^{-1}$ per year (*p*-value = 0.015) (Fig. 5e). We argue that global warming has contributed to an increase in VC over Tehran at night, particularly in the light that warming of Tehran has been much greater at night than during the day^27^. Despite that, due to a substantial increase in pollutant emissions, air pollution has rapidly increased in Tehran in recent years^9^. Both the average wind speed through PBL and VC have increased over Tehran during the day for the period 1991–2020, but none of them is statistically significant (Fig. 5d, f).

## Conclusions

Using the ERA5 and MERRA-2 data, we analyzed PBLH, the average wind speed through PBL, VC, and PM2.5 concentration over Tehran on the diurnal, monthly, and seasonal timescales averaged during the period 1991–2020. Both PBLH and VC over Tehran show substantial seasonal variations, with much higher values in summer followed in decreasing order by spring, autumn, and winter. In particular, air pollution potential is extremely high over Tehran in winter over the course of the day and night, particularly at night. Hence, due to the existence of pollutant emissions, the occurrence of severe air pollution is expected to be a common feature in Tehran during winter. Similar to our results^16^, found that the maximum VC over Hyderabad in India is during summer, while the minimum is in winter. It should be clarified that while our results indicate high air pollution potential in winter compared to summer over Tehran, PM2.5 concentration is much higher in summer than winter because Tehran is largely influenced by dust events in late spring and during summer^26^. Seasonal variation in the average wind speed through PBL over Tehran is small compared to seasonal variation in PBLH and VC because relatively stronger winds at winter nights compared to the other seasons partially compensate for the stronger winds during the day in warmer months of spring, summer, and autumn.

PBLH and VC also show substantial diurnal variations, with higher values during the day and much lower values at night. Indeed, air pollution potential over Tehran is the worst between 05:30 and 07:30 LT, while the best ventilation condition is between 13:30 and 14:30 LT. As a result, PM2.5 concentration is relatively lower over Tehran in the afternoon, while the maximum occurs in the early morning between 05:30 and 07:30 LT. This is consistent with the results of^5^ over Kochi in southwest India. They found that the ventilation of air pollutants is high in the afternoon, but reduces in the morning and evening. The average wind speed through PBL also shows the same diurnal variation in all seasons, except in winter when winds in PBL are stronger at night than during the day. Our analysis indicates that substantial ventilation of air pollutants occurs over Tehran after sunrise in warmer months of spring, summer, and autumn (particularly in summer), while the ventilation of air pollutants is poor both at night and during the day in winter.

We also analyzed trends of PBLH, the average wind speed through PBL, and VC during the period 1991–2020. PBLH has significantly increased over Tehran both during the day and at night for the period 1991–2020, primarily in response to the surface warming in recent decades^27^, while wind speed through PBL has significantly declined only at night. The overall impact of such changes is an increase in VC over Tehran both during the day and at night, although only the increasing trend of VC is statistically significant at night. Similarly^18^, also identified an increasing trend in VC over Delhi during the period 2006-2014. Despite the increasing trend of VC over Tehran, air pollution has rapidly increased in Tehran in recent years^9^ due to a substantial increase in pollutant emissions.

In this study, variations of VC over Tehran on different timescales are determined based on the analysis of PBLH and the average wind speed through PBL. Precipitation is another contributing factor that largely impacts air pollution potential due to the scavenging of air pollutants by precipitation. Hence, as precipitation is higher over Tehran from November to April^27^, it can partially contribute to the ventilation of air pollutants. Further investigation is required to understand the combined effect of PBLH, the average wind speed through PBL, and precipitation on the ventilation of air pollutants over Tehran.

The high air pollution potential over Tehran in winter identified in this study implies that the natural ventilation of air pollutants is extremely weak over Tehran in winter. The extremely poor air quality of Tehran in recent decades based on observations [e.g.^9^] indicates that the air pollution problem is rapidly growing in Tehran. Hence, effective environmentally sustainable planning and management are urgently required to reduce air pollution in Tehran, including improving energy efficiency, replacing energy sources that have larger effects on air pollution with renewable clean sources, reducing energy consumption, and relocating pollution-intensive industries.

## Data Availability

We obtained the ERA5 data from the ECMWF data server on pressure levels at https://cds.climate.copernicus.eu/cdsapp#!/dataset/reanalysis-era5-pressure-levels?tab=form and single levels at https://cds.climate.copernicus.eu/cdsapp#!/dataset/reanalysis-era5-single-levels?tab=form. We also obtained the MERRA-2 data from https://giovanni.gsfc.nasa.gov/giovanni/.

## Code availability

The datasets generated during and/or analyzed during the current study are available from the corresponding author on reasonable request.
